# Does oral iron and folate supplementation during pregnancy protect against adverse birth outcomes and reduced neonatal and infant mortality in Africa: A protocol for a systematic review and meta-analysis?

**DOI:** 10.1177/02601060241256200

**Published:** 2024-05-23

**Authors:** Yibeltal Bekele, Claire Gallagher, Mehak Batra, Melissa Buultjens, Senem Eren, Bircan Erbas

**Affiliations:** 1 110573 School of Psychology and Public Health, La Trobe University, Melbourne, VIC, Australia; 2 School of Public Health, Bahir Dar University, Bahir Dar, Ethiopia; 3 50066 School of Population and Global Health, The University of Melbourne, Melbourne, VIC, Australia; 4 School of Humanities and Social Sciences, Ibn Haldun University, Istanbul, Turkey

**Keywords:** Pregnant mother, iron supplementation, folate supplementation, iron folate supplementation, adverse birth outcomes, neonatal mortality, infant mortality, Africa

## Abstract

**Background:** Globally, one-third of pregnant women are at risk of iron deficiency, particularly in the African region. While recent findings show that iron and folate supplementation can lower the risk of adverse birth outcomes and childhood mortality, our understanding of its impact in Africa remains incomplete due to insufficient evidence. This protocol outlines the systematic review steps to investigate the impact of oral iron and folate supplementation during pregnancy on adverse birth outcomes, neonatal mortality and infant mortality in Africa. **Methods and analysis:** MEDLINE, PsycINFO, Embase, Scopus, CINAHL, Web of Science, and Cochrane databases were searched for published articles. Google Scholar and Advanced Google Search were used for gray literature and nonindexed articles. Oral iron and/or folate supplementation during pregnancy is the primary exposure. The review will focus on adverse birth outcomes, neonatal mortality and infant mortality. Both Cochrane Effective Practice and Organization of Care and Newcastle-Ottawa Scale risk of bias assessment tools will be used. Meta-analysis will be conducted if design and data analysis methodologies permit. This systematic review and meta-analysis will provide up-to-date evidence about iron and folate supplementation's role in adverse birth outcomes, neonatal mortality and infant mortality in the African region. **Ethics and dissemination:** This review will provide insights that help policymakers, program planners, researchers, and public health practitioners interested in working in the region. **PROSPERO registration number:** CRD42023452588.

## Introduction

Anemia during pregnancy is mainly caused by iron deficiencies and affects 37% of pregnant women globally (Lopez et al., 2016; World Health Organization (WHO), 2022). This burden is disproportionately borne by women in low-income countries, particularly in the region of Africa where 44.3% of pregnant women are anemic (Araujo Costa and de Paula Ayres-Silva, 2023). During pregnancy, anemia is associated with adverse birth outcomes (stillbirth, low birth weight, and preterm birth), perinatal, neonatal mortality and infant mortality (Bourassa et al., 2019; Caniglia et al., 2022; Oaks et al., 2019; Papadopoulou et al., 2013; Yeboah et al., 2022).

Each year, an estimated 19.8 million low birth weight births, 13.4 million preterm births, and 2.5 million neonatal deaths are reported worldwide (Hug et al., 2021, 2019; United Nations International Children's Emergency Fund (UNICEF), 2023). Over 80% of these cases occur in low and middle-income countries (LMICs) (Blencowe et al., 2019; Hug et al., 2021; Ohuma et al., 2023; Sharrow et al., 2022), with Africa alone accounting for 43% of global preterm births and 28% of stillbirths (Hug et al., 2021; Ohuma et al., 2023). When compared with high-income countries, disparities in maternal and child morbidity and mortality within the African region are particularly revealing (Blencowe et al., 2016, 2019; Ohuma et al., 2023). For example, cross-sectional studies conducted in Nigeria and Burundi reported that the rates of stillbirth were 39.6 and 23 per 1000 live births, respectively (Akombi et al., 2018; Okonofua et al., 2019), more than seven times higher than the rate in Western countries (2.9 per 1000 live births) (Hug et al., 2021). Another review in Ethiopia reported that the pooled prevalence of low birth weight was 17.3%, which is more than double the prevalence of low birth weight in Europe (ranging from 6.8% to 7.1%) (Blencowe et al., 2019; Endalamaw et al., 2018).

In the last 20 years, global rates of neonatal mortality, low birth weight, and stillbirths have decreased by 51%, 16.6%, and 25.5% respectively (Blencowe et al., 2019; Hug et al., 2019, 2021). However, progress in Africa, especially in the sub-Sahara region, has been slow (Blencowe et al., 2016, 2019; Hug et al., 2021). At this rate, African countries are unlikely to meet the Sustainable Development Goals aiming to reduce stillbirth and neonatal mortality rates to below 12% by 2030, along with the global target to decrease low birth weight by 30% by 2025 (United Nations Fund for Development (UNFD), 2015; WHO, 2017).

Various factors contribute to the ongoing challenges in Africa, including high poverty, poor healthcare financing, poor access to quality healthcare, poor utilization of recommended healthcare services, and widespread food insecurities (Ansu-Mensah et al., 2020; Mason, 2007; Wigley et al., 2020). A cross-sectional study conducted in Rwanda and Ethiopia reported that the uptake of adequate Antenatal Care (ANC) was 48.58% and 20% respectively (Negash et al., 2023; Uwimana et al., 2023), which is significantly lower than the global antenatal care coverage of 88% (UNICEF, 2023). Beyond limited access to ANC services, the quality of antenatal care services in Africa is poor. A study conducted in East Africa among 46,656 women reported that 11.16% of pregnant women received good-quality antenatal care services (Raru et al., 2022). Similarly, a study conducted in Ghana reported that only 7.6% of pregnant women who attended ANC received good-quality antenatal care services (Amponsah-Tabi et al., 2022).

Healthcare financing is the other main challenge in Africa. Country-specific reports released since 2018 from Ethiopia, Tanzania, and Burundi showed that the per capita healthcare expenditure of the countries was US$36.40 (Ministry of Health, 2022), US$36.80 (UNICEF, 2020), and US$8.67, respectively (Burundi Minister of Health, 2023). These figures fall well below half of the World Health Organization (WHO) recommended per capital expenditure of US$86 to ensure the delivery of essential healthcare services (Jowett et al., 2016). Therefore, implementing cost-effective interventions in the region is crucial for addressing the prevailing challenges.

To alleviate this burden, the WHO recommends that all pregnant women take daily iron and folic acid (IFA) supplementation as a cost-effective intervention to prevent adverse birth outcomes, perinatal mortality, neonatal mortality and infant mortality. Specifically, the revised antenatal care guidelines advise all pregnant women to take 30 to 60 mg of iron and 0.4 mg of folic acid daily (Tuncalp et al., 2020; WHO, 2016), as folic acid increases the uptake of iron and accelerates the resolution of anemia.

Despite this recommendation, the coverage and adherence to iron and folate supplementations are very low in Africa. For example, a recent cross-sectional study showed adherence to iron and iron folate supplementation in Africa ranged from 3.2% to 21.2% (Karyadi et al., 2023). Another large population-based study across 22 sub-Saharan African countries showed adherence to iron supplementation ranged from 1.4% in Burundi to 72% in Senegal (Ba et al., 2019). A cross-sectional study conducted in Nigeria and Tanzania reported that adherence to folate and combined iron folate supplementation was 36.5% and 20.3%, respectively (Ifeoluwapo et al., 2020; Lyoba et al., 2020). Similarly, a recent study conducted in Ethiopia showed adherence to iron and folate supplementation was 20% (Tamirat et al., 2022). As demonstrated by these and many other studies, nutrition-related problems continue to be a major public health challenge in Africa (Beyene, 2023; Militao et al., 2022).

Existing meta-analyses of randomized control trials (RCTs) and primary studies show iron and iron-folate supplementation during pregnancy is effective in reducing the risk of maternal anemia (Oh et al., 2020; Peña-Rosas et al., 2015), preterm birth (Haider et al., 2013; Iqbal and Ekmekcioglu, 2019), low birth weight (Haider et al., 2013; Iqbal and Ekmekcioglu, 2019; Nisar and Dibley, 2016; Oh et al., 2020; Tadesse et al., 2023), prenatal mortality (Yeboah et al., 2022), neonatal mortality (Nisar and Dibley, 2016; Rai et al., 2022; Titaley and Dibley, 2012) and infant mortality (Dibley et al., 2012). The doses of iron, folate, and combined supplementations are clinically important to determine birth outcomes and child survival but there is variation in the supplementation doses across studies (Papadopoulou et al., 2013; West et al., 2014) even though the WHO recommended daily iron folate supplementation containing 30 to 60 mg of iron and 0.4 mg of folic acid (WHO, 2016) is considered effective in improving birth and newborn health.

Several systematic reviews of LMIC have previously explored the effectiveness of supplementation during pregnancy on varied outcomes of perinatal morbidity and/or mortality (Bourassa et al., 2019; Haider et al., 2013; Imdad and Bhutta, 2012; Mahomed, 2000; Oh et al., 2020; Smith et al., 2017). However, only a proportion of the included studies were conducted in the African region, and half of the reviews focused exclusively on the effectiveness of multiple micronutrient supplementation rather than iron and/or folate supplementation, which is the main nutritional supplement provided in routine antenatal care practice in the African region (Ethiopian Public Health Institute (EPHI)[Ethiopia] and ICF, 2019; ICF., 2021; National Population Commission (NPC) [Nigeria] and ICF International, 2019; National Institute of Statistics of Rwanda (NISR), 2021; Rakanita et al., 2021). Furthermore, while the other reviews provide evidence of the effectiveness of iron and/or folate supplementation, they were conducted more than a decade ago and reported inconsistent findings (Haider et al., 2013; Imdad and Bhutta, 2012; Mahomed, 2000). The 2000 review showed iron and folate supplementation had no detectable impact on birth outcomes (such as preterm delivery, low birth weight, and stillbirth/neonatal death) (Mahomed, 2000). Whereas, 2012 (Imdad and Bhutta, 2012) and 2013 (Haider et al., 2013) reviews found that iron alone or with folic acid supplementation reduced the risk of low birth weight but had no effect on preterm birth, small for gestational age and perinatal mortality (Imdad and Bhutta, 2012). Haider et al. (2013) also assessed the effect of dose response on adverse birth outcomes, finding that increasing the daily iron dose by 10 mg with a maximum of 66 mg reduces the risk of low birth weight and the dose response did not affect preterm birth.

Although LMICs face common challenges related to poor health, it is important to recognize their inherent heterogeneity. These countries exhibit diverse racial, ethnic, and contextual factors, which contribute to the complexity of their health inequalities. In this regard, the effectiveness of iron and/or folate supplementation may differ across regions, highlighting the value of synthesizing region-specific evidence. Accordingly, the present study seeks to update current evidence on the effectiveness of iron and/or folate supplementation during pregnancy on perinatal morbidity and mortality by conducting a comprehensive review of African studies that incorporates recently published articles. While previous reviews have mostly been interested in experimental studies, our synthesis will also encompass studies of observation design. Although observational studies are lower in the hierarchy of evidence, resource-poor settings like Africa often lack the financial and human capacity to conduct experimental studies (Alemayehu et al., 2018). Therefore, we have deliberately included observational studies, so as not to limit the availability of evidence in this region.

To our knowledge, this systematic review will be the first to synthesize evidence from both interventional and observational studies to summarize the effectiveness of oral iron and/or folate supplementation during pregnancy in protecting against adverse birth outcomes and neonatal mortality and infant mortality in Africa. Our specific objectives are:

1. Synthesize the available evidence on associations between oral iron and/or folate supplementation and adverse birth outcomes, neonatal mortality and infant mortality.
2. Evaluate the dose–effect and duration of oral iron and/or folate supplementation on adverse birth outcomes, neonatal mortality and infant mortality in Africa.
3. Investigate urban–rural differences in the effects of oral iron and/or folate supplementation on adverse birth outcomes, neonatal mortality and infant mortality in Africa.
4. If the data permits, a stratified analysis will be conducted on regional differences.

## Methods

This review protocol was written following the Preferred Reporting Items for Systematic Review and Meta-Analysis Protocol (PRISMA-P) 2015 checklist (Moher et al., 2015) (see checklist in Supplemental File 1). In reporting and synthesizing the results of the systematic review, we will follow the PRISMA 2020 guidelines (Page et al., 2021). This protocol was prospectively registered with PROSPERO (Reg No.: CRD42023452588).

### Research question

This review will seek to answer the following research questions.

1. Does iron-only, folate-only, and IFA supplementation during pregnancy protect against adverse birth outcomes and reduced neonatal mortality and infant mortality in Africa compared to pregnant women who were administered a placebo or did not receive any supplementation?
2. Does the dosage and duration of supplementation influence the likelihood of adverse birth outcomes, neonatal mortality and infant mortality in Africa?

### Eligibility criteria

#### Types of studies

Quantitative studies of either intervention or observational design will be considered, including randomized and quasi-RCTs, cohort, case-control, and cross-sectional studies. This review will exclude abstracts without full articles, conference papers, case reports, and case series.

#### Population of interest

This study will include pregnant women of any age, gestation, or parity living in Africa.

#### Exposure

The exposure of interest is oral iron-only, folate-only, or combined IFA supplementation during pregnancy, regardless of the gestational age at which supplementation began, the duration of supplementation, or the dosage administered. Studies measuring supplementation via self-report, from maternal health records, or directly by researchers will be considered. Studies that involved the supplementation of IFA through fortification, or in combination with other micronutrients, will not be included. Studies that considered serum iron or folate levels as exposure will be excluded. We chose to exclude studies involving fortification to enhance comparability and minimize confounding factors. By focusing solely on oral iron-only, folate-only, and IFA supplementation, we aim to provide a clearer understanding of the isolated effects of these specific interventions. Furthermore, our interest also lies in exploring the dose–effect response of these supplementation approaches.

#### Comparators

The comparison groups under consideration will consist of pregnant women who were either administered a placebo or did not receive any supplementation.

#### Outcomes

##### Birth outcomes

- Stillbirth (termination of pregnancy after 28 weeks of gestation)
- Preterm birth (birth of the baby before 37 completed weeks)
- Small for gestational age (<10th percentile of weight for gestational age)
- Low birth weight (birth weight <2500 g) (Caniglia et al., 2022; Villar et al., 2014).

##### Infant outcomes

- Perinatal mortality (death between 28 weeks of gestation and first week of the neonatal period)
- Neonatal mortality (death of the newborn within the first 28 days of life)
- Infant mortality (infant death before the first birthday ≤365 days) (Caniglia et al., 2022; Villar et al., 2014).

#### Context and language

The inclusion criteria for this review are limited to studies conducted in Africa, with no restrictions on the time period. Additionally, articles published in languages other than English will be excluded from the scope of the review.

### Information sources

The databases, MEDLINE, PsycINFO, Embase, Scopus, CINAHL, Web of Science, and Cochrane were searched. Gray literature was searched using Google Scholar. Google Scholar and Google Advanced Search were used to identify relevant articles not indexed by databases and that may not be captured in the search strategy.

### Search strategy

The search strategy was developed in collaboration with the Latrobe University research librarian and is available in the online supplement (Supplemental Material File 2). Databases were searched on 29 August 2023 and results were limited to studies published in English. A total of 3349 articles were retrieved, and 1175 were excluded due to duplication. The final search of the databases will be conducted just before the submission of the manuscript.

### Study selection

**Figure 1.**
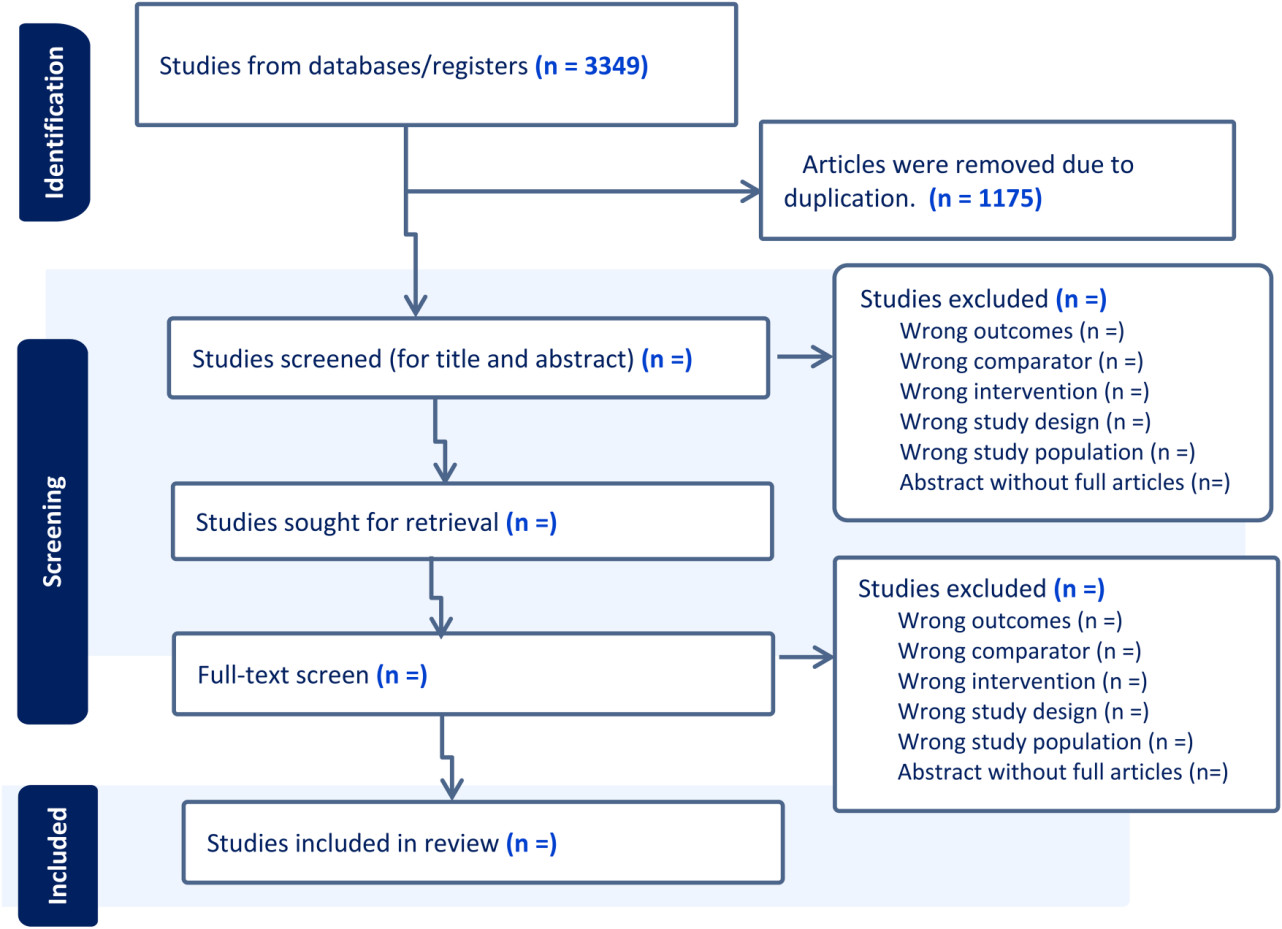
Preferred reporting items for systematic review and meta-analysis (PRISMA) flowchart diagram for study selection (Moher et al., 2015).

The search results from each database will be imported to Covidence for screening and data extraction. Covidence is a web-based software that assists in screening the title, abstract, and full title (Veritas Health Innovation, 2023). All duplicated articles will be removed. Two authors (YB and CG) will independently screen the titles and abstracts. Following this, the authors will independently review full-text articles based on the inclusion and exclusion criteria. Articles that fulfill the eligibility criteria will be included for final data extraction (Figure 1). Any disagreement between authors will be resolved by discussion or consultation with the senior author (BE).

### Data extraction

Data extraction from included studies will be conducted using a predesign standardized spreadsheet. Before the actual data extraction, the data extraction spreadsheet will be piloted on five randomly selected articles. Data extraction will be conducted independently by YB and CG. During data extraction names of authors, publication date, study setting, study design, study population, sample size, response rate, types of exposure, dosage of the exposure, duration of the exposure, measurement of /exposure, types of outcomes, confounder adjustment and effect estimates and corresponding 95% Confidence Interval will be extracted from all included articles.

### Quality appraisal

The quality of the published articles will be assessed using both Cochrane Effective Practice and Organizational Care (EPOC) (Cochrane Effective Practice and Organization of Care (EPOC), 2017) and the Newcastle-Ottawa Scale (NOS) risk of bias assessment tool (Peterson et al., 2011). Two authors will separately assign and review each article using a risk of bias assessment tool. EPOC risk of bias assessment tool will be used to evaluate the quality of published articles of experimental and quasi-experimental study design. Then based on the EPOC risk of bias assessment tool criteria, each article will be ranked low, high, and unidentified risk of bias. The quality of observational studies such as cohort, case-control, and cross-sectional study design will be assessed by using NOS risk of bias assessment tools and finally, each article will be ranked low, medium, and high risk of bias. Any difference in rating between authors will be resolved by discussion with the senior author (BE).

### Data synthesize and meta-analysis

All articles will be grouped based on their exposure and reported outcome/s (stillbirth, preterm birth, small for gestational age, low birth weight, neonatal mortality and infant mortality). For studies that report multiple outcomes, the results will be presented separately, under each outcome.

If the available study designs and data analysis from each study allow, we will conduct a meta-analysis for the exposures and each outcome stratified by country. The estimated effect sizes, standard errors, and corresponding 95% CI will be extracted. Variables used as confounders or effect modifications will also be extracted to better understand the methodologies implemented in each study thereby guiding the applicability of the meta-analysis frameworks. Meta-analysis will be conducted using Stata Version 18. Statistical heterogeneity among studies will be assessed using the Q and I2 statistics. Funnel plots with Egger or Begg rank correlation tests will be used for publication bias. Random-effect models will be used in all meta-analyses. Time points (duration of exposure) are to be considered in the meta-regressions and other factors such as rurality, comorbidity, and country-specific effects may be important for subgroup analysis. If the data does not permit meta-analysis we will conduct a narrative synthesis guided by the Synthesis Without Meta-analysis reporting guidelines (Campbell et al., 2020).

## Discussion

This protocol details the methods for systematically locating and synthesizing the available evidence on the effects of oral iron and/or folate supplementations on birth outcomes, neonatal mortality and infant mortality among African childbearing women, and their offspring. Evidence sourced from peer-reviewed and gray literature will be qualitatively synthesized and if data permits, a meta-analysis will be conducted to quantify overall effect estimates.

Previously, reviews conducted in LMIC and other parts of the world focused on evaluating the effectiveness of multiple micronutrient supplementations compared to other micronutrient supplementations (such as iron or iron folate) (Haider et al., 2011; Oh et al., 2020; Smith et al., 2017) and not on oral iron and/or folate supplementation. Moreover, few reviews assessed the dose-response effect of these supplements on outcomes as described in our protocol (Haider et al., 2013). Such information will be immensely useful for the development of cost-effective interventions, especially in rural and remote regions.

Additionally, while iron and folate supplementation is recommended for all pregnant women (WHO, 2016), further synthesis of evidence is required to evaluate the effectiveness of iron and folate supplementation, in anemic prevalent areas, such as Africa, with varying urban and rural population characteristics (Tuncalp et al., 2020). Africa, particularly sub-Saharan Africa, does have a high prevalence of anemia (Mwangi et al., 2021) and more than half of the population does not have access and quality primary healthcare services (Cullinan, 2021). Accordingly, poor birth outcomes, neonatal mortality and infant mortality remain substantial challenges in the region. Specifically, evidence indicated that Africa records 20.5–22.8 stillbirths per 1000 live births (Hug et al., 2021), and accounts for 29% of all preterm births and 46% of all neonatal deaths reported globally (McAllister et al., 2019). The burden of maternal and child morbidity and mortality is particularly high in rural areas, where the coverage of and adherence to iron supplementation are much lower than in urban areas (Diallo et al., 2012; Shibre et al., 2020).

A report in Ethiopia showed that 54.9% of pregnant women living in rural areas have taken iron supplementation but only 9% of pregnant women adhere to the recommended days (Ethiopian Public Health Institute (EPHI)[Ethiopia] and ICF, 2019). Similarly report in Nigeria showed around 48% of pregnant women living in rural areas have taken iron supplementation but only 25% of pregnant women adhere to iron supplementation (National Population Commission (NPC)[Nigeria] and ICF International, 2019) which is much lower compared to the urban residents. Through the methods outlined in this protocol, this systematic review and meta-analysis seeks to address the gaps in the current literature to allow for a more comprehensive understanding of the effect of iron and/or folate supplementation on adverse birth and child health outcomes.

By addressing these critical research gaps, the findings of this systematic review can be instrumental for policymakers, programmers, researchers, and public health practitioners who are interested in working in the region to understand the impacts of iron and folate supplementation on reducing adverse birth outcomes, neonatal mortality and infant mortality in Africa. For instance, policymakers can utilize the evidence-based recommendations from this review to shape policies and interventions aimed at improving maternal and child health outcomes through targeted supplementation strategies. Programmers can also utilize the findings to design and implement effective interventions tailored to specific populations and settings, considering factors such as supplementation duration, dosage, and disparities in rural or remote areas.

## Supplemental Material

sj-doc-1-nah-10.1177_02601060241256200 - Supplemental material for Does oral iron and folate supplementation during pregnancy protect against adverse birth outcomes and reduced neonatal and infant mortality in Africa: A protocol for a systematic review and meta-analysis? Supplemental material, sj-doc-1-nah-10.1177_02601060241256200 for Does oral iron and folate supplementation during pregnancy protect against adverse birth outcomes and reduced neonatal and infant mortality in Africa: A protocol for a systematic review and meta-analysis? by Yibeltal Bekele, Claire Gallagher, Mehak Batra, Melissa Buultjens, Senem Eren and Bircan Erbas in Nutrition and Health

## List of Abbreviations

ANC: Antenatal Care
EPOC: Cochrane effective practice and organization of care
LMICs: Low- and middle-income countries
Mg: Milligram
NOS: Newcastle-Ottawa Scale
PRISMA-P: Preferred reporting items for systematic review and meta-analysis protocol
RCTs: Randomized control trials
SWiM: Synthesis without meta-analysis
US: United States
WHO: World Health Organization
