# Supplementary material for: Does oral iron and folate supplementation during pregnancy protect against adverse birth outcomes and reduced neonatal and infant mortality in Africa: A protocol for a systematic review and meta-analysis?
Source: Nutr Health. 2024 May 23;31(1):15–22. doi: 10.1177/02601060241256200 (PMC11954406; doi:10.1177/02601060241256200)
Supplement: sj-doc-1-nah-10.1177_02601060241256200 - Supplemental material for Does oral iron and folate supplementation during pregnancy protect against adverse birth outcomes and reduced neonatal and infant mortality in Africa: A protocol for a systematic review and meta-analysis? [file sj-doc-1-nah-10.1177_02601060241256200.doc]

**Supplementary file 1:** PRISMA-P checklist of a systematic review protocol

| Section and topic | Item No | Page numbers | Checklist item |
| --- | --- | --- | --- |
| ADMINISTRATIVE INFORMATION | | | |
| Title: |  |  |  |
| Identification | 1a | Title page | Identify the report as a protocol of a systematic review |
| Update | 1b | Not applicable | If the protocol is for an update of a previous systematic review, identify as such |
| Registration | 2 | Title page | If registered, provide the name of the registry (such as PROSPERO) and registration number |
| Authors: |  |  |  |
| Contact | 3a | Title page | Provide name, institutional affiliation, e-mail address of all protocol authors; provide physical mailing address of corresponding author |
| Contributions | 3b | Title page | Describe contributions of protocol authors and identify the guarantor of the review |
| Amendments | 4 | Not applicable | If the protocol represents an amendment of a previously completed or published protocol, identify as such and list changes; otherwise, state plan for documenting important protocol amendments |
| Support: |  |  |  |
| Sources | 5a | Not applicable | Indicate sources of financial or other support for the review |
| Sponsor | 5b | Title page | Provide name for the review funder and/or sponsor |
| Role of sponsor or funder | 5c | Not applicable | Describe roles of funder(s), sponsor(s), and/or institution(s), if any, in developing the protocol |
| INTRODUCTION | | | |
| Rationale | 6 | 2 - 5 | Describe the rationale for the review in the context of what is already known |
| Objectives | 7 | 5 | Provide an explicit statement of the question(s) the review will address with reference to participants, interventions, comparators, and outcomes (PICO) |
| METHODS | | | |
| Eligibility criteria | 8 | 5-6 | Specify the study characteristics (such as PICO, study design, setting, time frame) and report characteristics (such as years considered, language, publication status) to be used as criteria for eligibility for the review |
| Information sources | 9 | 6-7 | Describe all intended information sources (such as electronic databases, contact with study authors, trial registers or other grey literature sources) with planned dates of coverage |
| Search strategy | 10 | 7 | Present draft of search strategy to be used for at least one electronic database, including planned limits, such that it could be repeated |
| Study records: |  | |  |
| Data management | 11a | 6-7 | Describe the mechanism(s) that will be used to manage records and data throughout the review |
| Selection process | 11b | 7 | State the process that will be used for selecting studies (such as two independent reviewers) through each phase of the review (that is, screening, eligibility and inclusion in meta-analysis) |
| Data collection process | 11c | 8 | Describe planned method of extracting data from reports (such as piloting forms, done independently, in duplicate), any processes for obtaining and confirming data from investigators |
| Data items | 12 | 6 | List and define all variables for which data will be sought (such as PICO items, funding sources), any pre-planned data assumptions and simplifications |
| Outcomes and prioritization | 13 | 6 | List and define all outcomes for which data will be sought, including prioritization of main and additional outcomes, with rationale |
| Risk of bias in individual studies | 14 | 7-8 | Describe anticipated methods for assessing risk of bias of individual studies, including whether this will be done at the outcome or study level, or both; state how this information will be used in data synthesis |
| Data synthesis | 15a | 8 | Describe criteria under which study data will be quantitatively synthesised |
|  | 15b | 8 | If data are appropriate for quantitative synthesis, describe planned summary measures, methods of handling data and methods of combining data from studies, including any planned exploration of consistency (such as I^2^, Kendall’s τ) |
|  | 15c | 8 | Describe any proposed additional analyses (such as sensitivity or subgroup analyses, meta-regression) |
|  | 15d | 8 | If quantitative synthesis is not appropriate, describe the type of summary planned |
| Meta-bias(es) | 16 | 8 | Specify any planned assessment of meta-bias(es) (such as publication bias across studies, selective reporting within studies) |
| Confidence in cumulative evidence | 17 | Not applicable | Describe how the strength of the body of evidence will be assessed (such as GRADE) |

**Supplementary file 2: Preliminary search strategy from MEDLINE.**

| **No.** | **Concept** | **Search terms** |
| --- | --- | --- |
| 1 | Pregnant women | pregnancy/ or exp gravidity/ or exp labor, obstetric/ or exp parity/ or exp parturition/ or exp placentation/ or exp pregnancy, multiple/ or exp pregnancy, twin/ or exp superfetation/ or exp pregnancy, unplanned/ or exp pregnancy, unwanted/ or Pregnant Women/or mothers/ or exp single parent/ or exp surrogate mothers/ or adolescent mothers/ or perinatal care/ or exp postnatal care/ or exp preconception care/ or exp prenatal care/ or (pregnan* or wom#n* or mother* or mom* or matern* or partus or lactation or labo#r or childbear* or child-bear* or gestation* or antenatal or ante-natal or "ante natal" or pre-natal or prenatal or peri-natal or perinatal or peri-partum or "peri partum" or "child birth" or child-birth or childbirth or "term birth") |
| 2 | Iron folate supplementation | Micronutrients supp/ or exp trace elements/ or exp iron/ or exp vitamins/ or exp folic acid/ or ("Iron folate supplementation" or "Iron and folate supplementation" or "iron supplementation" or "folic acid supplementation") |
| 3 | Adverse birth outcomes/neonatal /infant mortality | pregnancy complications/ or exp abortion, spontaneous/ or exp abortion, habitual/ or exp abortion, incomplete/ or exp abortion, missed/ or exp abortion, septic/ or exp abortion, threatened/ or exp fetal death/ or exp stillbirth/ or exp perinatal death/ or infant/ or exp infant, newborn/ or exp infant, low birth weight/ or exp infant, small for gestational age/ or exp infant, very low birth weight/ or exp infant, extremely low birth weight/ or exp infant, postmature/ or exp infant, premature/ or exp infant, extremely premature/ or infant mortality/ or ("pregnanc* complication*" or "low birth weight" or stillbirth or "still birth" or "low birth weight" or "preterm birth" or miscarriage or abortion or "prenatal mortality" or "perinatal mortality" or "very low small for weight" or "small for gestational age" or "newborn death" or "early neonatal mortality" or "late neonatal mortality" or "post neonatal mortality" or "neonatal mortality" or "post neonatal mortality" or "infant mortality").mp. |
| 4 | Africa | Africa/ or exp africa, northern/ or exp algeria/ or exp egypt/ or exp libya/ or exp morocco/ or exp tunisia/ or exp "africa south of the sahara"/ or exp africa, central/ or exp cameroon/ or exp central african republic/ or exp chad/ or exp congo/ or exp "democratic republic of the congo"/ or exp equatorial guinea/ or exp gabon/ or exp "sao tome and principe"/ or exp africa, eastern/ or exp burundi/ or exp comoros/ or exp djibouti/ or exp eritrea/ or exp ethiopia/ or exp kenya/ or exp madagascar/ or exp rwanda/ or exp seychelles/ or exp somalia/ or exp south sudan/ or exp sudan/ or exp tanzania/ or exp uganda/ or exp africa, southern/ or exp angola/ or exp botswana/ or exp eswatini/ or exp lesotho/ or exp malawi/ or exp mozambique/ or exp namibia/ or exp south africa/ or exp zambia/ or exp zimbabwe/ or exp africa, western/ or exp benin/ or exp burkina faso/ or exp cabo verde/ or exp cote d'ivoire/ or exp gambia/ or exp ghana/ or exp guinea/ or exp guinea-bissau/ or exp liberia/ or exp mali/ or exp mauritania/ or exp niger/ or exp nigeria/ or exp senegal/ or exp sierra leone/ or exp togo/ or ("Sub Sahara Africa" or Africa or "central Africa" or "western Africa" or "east Africa" or "southern Africa" or "Norther Africa" or "Burkina Faso" or Burundi or "Central Africa Republic" or Chad or "DR Congo" or Eritrea or Ethiopia or Gambia or Guinea or Guinea-bisseau or Liberia or Madagascar or Malawi or Mali or Mozambique or Niger or Rwanda or "Sierra Leone" or Somalia or "South Sudan" or Sudan or Togo or Uganda or Zambia or Algeria or Angola or Benin or Cameroon or "Cape Verde" or Comoros or Djibouti or Egypt or Eswatini or Ghana or "Ivory Coast" or Kenya or Lesotho or Mauritania or Morocco or Nigeria or "Republic of the Congo" or Senegal or Tanzania or Tunisia or Zimbabwe or Botswana or "Equatorial Guinea" or Gabon or Libya or Mauritius or Namibia or "South Africa") |
| **5** | Combination | 1 AND 2 AND 3 AND 4 |
